# Comparative analysis of photon-counting and energy-integrating detector CT to identify obstructive coronary artery disease

**DOI:** 10.1007/s00330-025-12118-7

**Published:** 2025-11-14

**Authors:** Melinda Boussoussou, Milán Vecsey-Nagy, Zsófia Jokkel, Borbála Vattay, Anikó Kubovje, Barbara Sipos, Márton Kolossváry, Anikó Ilona Nagy, Lili Száraz, Sámuel Beke, Bernard Schmidt, Máté Kiss, Béla Merkely, Josua A. Decker, Tilman Emrich, Akos Varga-Szemes, Pál Maurovich-Horvat, Bálint Szilveszter

**Affiliations:** 1 Semmelweis University Heart and Vascular Center, Budapest, Hungary; 2 Department of Radiology and Radiological Science29425, Medical University of South Carolina, Charleston, SC USA; 3 Department of Radiology, Medical Imaging Centre, Semmelweis University, Budapest, Hungary; 4 Gottsegen National Cardiovascular Center, Budapest, Hungary; 5 Physiological Controls Research Center, University Research and Innovation Center, Óbuda University, Budapest, Hungary; 6 Siemens Healthineers AG, Forchheim, Germany; 7 Friedrich-Alexander-University Erlangen, Erlangen, Germany; 8 Siemens Healthcare, Budapest, Hungary; 9 Department of Diagnostic and Interventional Radiology, University Hospital Augsburg, Augsburg, Germany; 10 Department of Diagnostic and Interventional Radiology, University Medical Center of the Johannes Gutenberg-University, Mainz, Germany; 11 German Centre for Cardiovascular Research, Partner site Rhine-Main, Mainz, Germany

**Keywords:** Photon-counting detector CT, Invasive coronary angiography, Coronary stenosis, Diagnostic accuracy, Dual source CT

## Abstract

**Objective:**

To evaluate the patient-, vessel- and segment-based diagnostic performance of photon-counting detector CT (PCD-CT) compared to energy-integrating detector CT (EID-CT) for detecting ≥ 50% or ≥ 70% stenosis using invasive coronary angiography (ICA) as a reference standard.

**Materials and methods:**

Patients with stable chest pain and ≥ 50% stenosis detected on dual source PCD-CT who subsequently underwent ICA were prospectively enroled. Diagnostic accuracy was calculated for PCD-CT vs ICA and additionally for a patient cohort scanned with EID-CT with similar risk profiles and disease prevalence. A Monte Carlo simulation based on diagnostic accuracy parameters was performed to estimate the potential reduction in ICA referrals.

**Results:**

A total of 143 patients (66 ± 9 years, 27.3% female) with 572 vessels and 2431 segments were evaluated with PCD-CT and ICA. Regarding EID-CT, 109 patients (65 ± 9 years, 31.0% female), 436 vessels and 1853 segments were assessed, with every patient undergoing ICA. PCD-CT demonstrated significantly higher accuracy than EID-CT in detecting ≥ 50% stenosis: 88.1% vs 77.9% (patient level), 91.6% vs 77.8% (vessel level), and 97.7% vs 92.4% (segment level) (*p* < 0.01 for all). For detecting ≥ 70% stenosis, PCD-CT also showed higher accuracy than EID-CT: 90.9% vs 70.6% (patient level), 94.6% vs 80.9% (vessel level), and 98.6% vs 94.1% (segment level) (*p* < 0.01 for all). We demonstrated a potential mean reduction of 14.8% in ICA referrals when utilising PCD-CT compared to EID-CT.

**Conclusions:**

PCD-CT provides improved per-patient, per-vessel and per-segment diagnostic performance in detecting obstructive CAD in symptomatic patients when compared to patients scanned on EID-CT. PCD-CT may lead to a significant decrease in ICA utilisation.

**Key Points:**

***Question*** *Accurate coronary CT angiography guides treatment, but its diagnostic accuracy is limited by various factors*.

***Findings*** *Photon counting detector (PCD)-CT improved diagnostic performance in detecting ≥ 50% or ≥ 70% stenosis, potentially reducing unnecessary ICA referrals by 14.8%*.

***Clinical relevance*** *PCD-CT improves diagnostic accuracy over EID-CT and may reduce unnecessary ICA*.

**Graphical Abstract:**

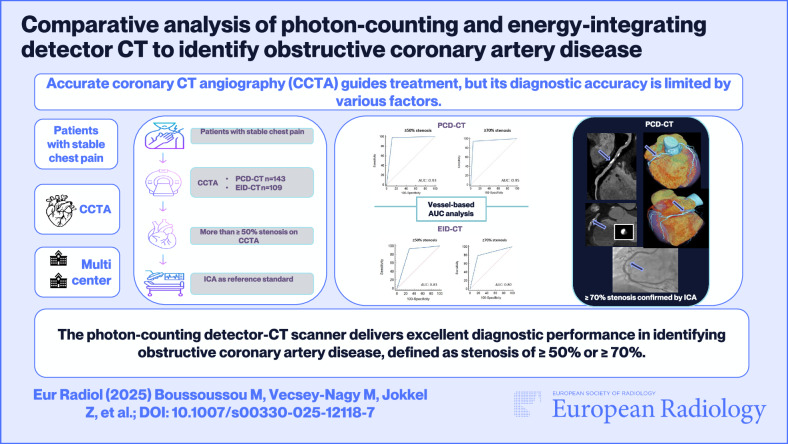

## Introduction

Both American and European guidelines recommend coronary CT angiography (CCTA) as the first-line test for assessing coronary artery disease (CAD) in symptomatic patients with low to moderate clinical likelihood of obstructive CAD [1–4]. Based on the DISCHARGE (Diagnostic Imaging Strategies for Patients with Stable Chest Pain and Intermediate Risk of Coronary Artery Disease) trial, CCTA-based care pathways are a safe alternative to invasive coronary angiography (ICA) with lower rates of periprocedural complications but similar rates of major adverse cardiac events [5]. Therefore, the number of CCTAs is expected to increase further, with higher-risk patients being increasingly referred for CCTA who may have extensive calcification or CAD burden [6].

Energy-integrating detector (EID)-CTs are hindered by limited spatial resolution, increased image noise, or blooming artefacts in patients with diffuse coronary calcification, often leading to an overestimation of coronary stenosis [7–9]. Photon-counting detector (PCD)-CT technology promises to address the above-mentioned limitations of EID-CTs [10–13]. PCD-CT enables the direct conversion of incoming photons to electrical signals and has the potential to provide CT data with higher spatial resolution, inherent spectral information, and improved noise characteristics. In addition, ultra-high resolution (UHR) mode is also available to achieve a maximum spatial in-plane image resolution of 0.11 mm, up to 40 lp/cm [12, 14, 15]. Exploiting the technical capabilities of PCD-CT, the first clinical evaluations demonstrated excellent image quality (IQ) [12, 16], more accurate assessment of luminal stenosis and stent patency [17–21], and reduced blooming in the presence of calcifications [22, 23] that could ultimately result in improved diagnostic accuracy [24, 25]. However, there is currently limited data regarding the diagnostic performance of the first-generation dual-source PCD-CT system in symptomatic patients referred for CCTA [23]. Therefore, our study aimed to evaluate the diagnostic accuracy of dual-source PCD-CT compared to EID-CT for detecting ≥ 50% or ≥ 70% stenosis in symptomatic patients referred for CCTA, using ICA as a reference standard.

## Materials and methods

### Study design

Our multicentric study involves two cohorts of consecutive, symptomatic patients referred for clinically indicated CCTA using either PCD-CT or EID-CT, at three tertiary cardiovascular centres (Semmelweis University Heart and Vascular Centre, Hungary, Semmelweis University Medical Imaging Centre, Hungary and Medical University of South Carolina, USA).

All patients underwent standard cardiac assessment-including ECG and laboratory tests-as part of their initial clinical evaluation. Individuals presenting with acute coronary syndromes, such as NSTEMI or unstable angina, were excluded from the study. CCTA was performed in patients with stable chest pain or angina-equivalent symptoms and low to intermediate risk factor-weighted clinical likelihood of obstructive CAD, in line with guideline-based indications [4].

**Fig. 1:**
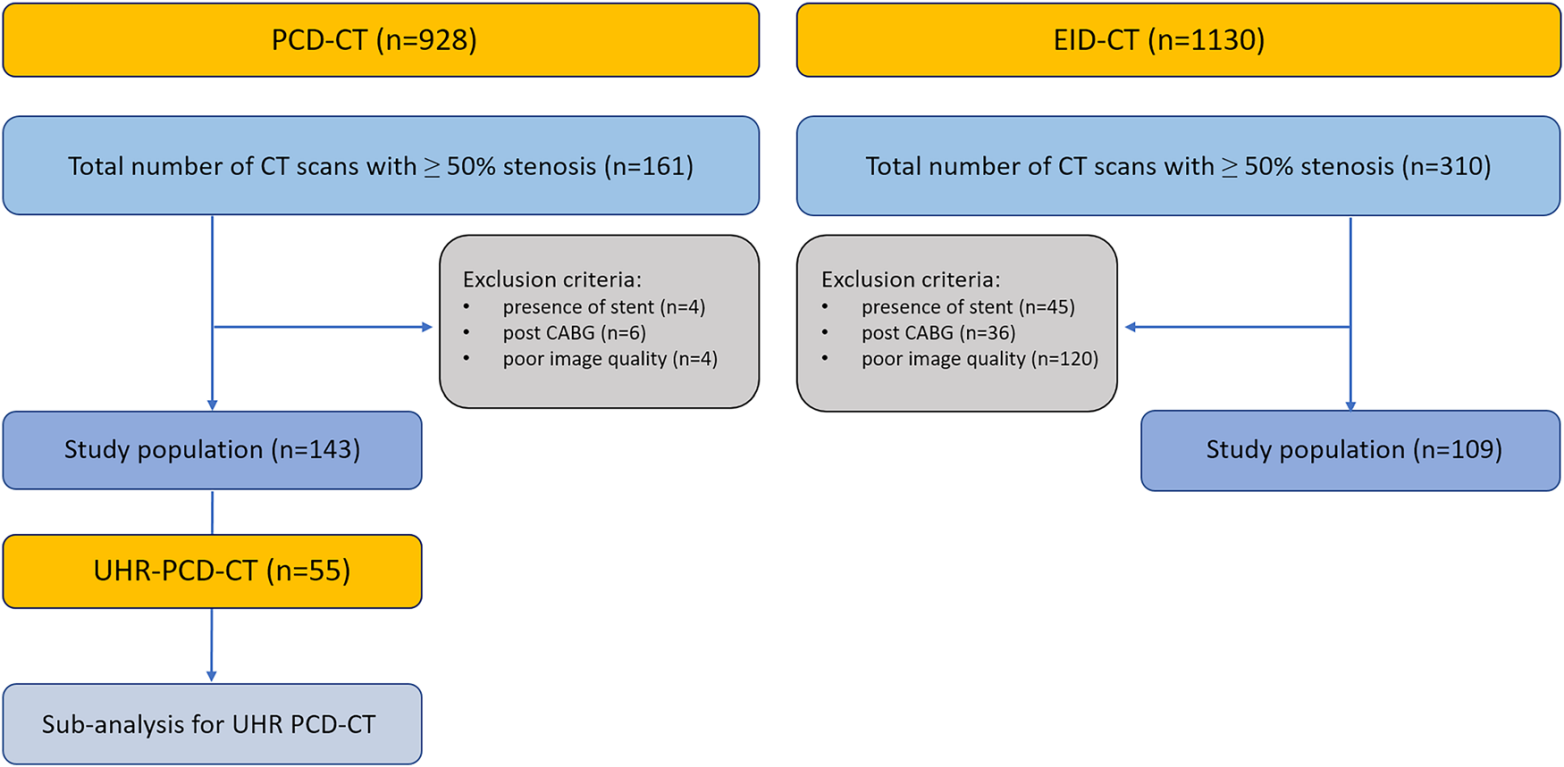
Study flow chart. The flow chart summarises the three patient populations scanned with either PCD-CT or EID-CT. A total of 143 patients were included in the final population scanned with PCD-CT, and an additional subanalysis was performed, limited to 55 patients who underwent UHR PCD-CT scan. The second additional cohort included 109 EID-CT patients after exclusion. PCD-CT scans were obtained from the Medical Imaging Centre at Semmelweis University and the Medical University of South Carolina, whereas EID-CT scans were acquired at the Heart and Vascular Centre, Semmelweis University. CABG, coronary artery bypass graft; PCD-CT, photon-counting detector computed tomography; UHR PCD-CT, ultra-high resolution photon-counting detector computed tomography; EID-CT, energy-integrating detector computed tomography

We retrospectively identified 2058 consecutive patients who underwent CCTA between June 1 and December 31, 2023, on a PCD-CT, or between January 1 and December 31, 2023, on an EID-CT system. PCD-CT scans were obtained from the Medical Imaging Centre at Semmelweis University and the Medical University of South Carolina, whereas EID-CT scans were acquired at the Heart and Vascular Centre of Semmelweis University. Inclusion criteria for the analysis in both cohorts were: (1) at least 18 years of age; (2) at least one coronary artery stenosis with ≥ 50% severity based on the CCTA report; and (3) ICA performed within 2 months after CCTA. Exclusion criteria across all cohorts were the following: (1) presence of a stent; (2) presence of a coronary artery bypass graft; and (3) poor IQ due to motion, step, breathing, or beam-hardening artefacts. The flow chart of the study is shown in Fig. 1. Baseline demographics, including anthropometrics and cardiovascular risk factors, were assessed using patient questionnaires at the time of the CCTA imaging.

Written informed consent was waived due to the retrospective nature of the analysis. The study was approved by local institutional and national ethical committees (IV/655-3/2022/EKU and 28803-5/2020/EÜIG) and performed in accordance with the Declaration of Helsinki.

### PCD-CT acquisition protocol

CCTA acquisition followed the guidelines of the Society of Cardiovascular Computed Tomography [26]. The sites generally adhered to standardised imaging protocols for cardiac scans. The same first-generation dual-source PCD-CT system (NAEOTOM Alpha, software version VB10, Siemens Healthineers) was used in the PCD-CT group on all sites. For non-contrast scans, a 120 kVp tube voltage was used. Sequential scan mode was primarily used for patients with heart rates < 65 bpm, while retrospective ECG-gated helical mode was applied in cases with higher or irregular heart rates. Regarding heart rate control, all patients without contraindications received oral or intravenous beta-blockers. All patients without contraindication received 0.4–0.8 mg of sublingual nitroglycerin or a 10 mg/24-h nitroglycerin transdermal patch to achieve proper vasodilation during CCTA. Injection protocol with bolus tracking was implemented at all sites, administering an optimised contrast volume at a flow rate of 4.5–6.0 mL/s endorsed by the Society of Cardiovascular Computed Tomography. Based on patients’ anthropometrics, the following scan parameters were set for CCTA: Tube voltage of 120 or 140 kVp (120 kvp for UHR), automatic tube current modulation, a detector configuration of 144 mm × 0.4 mm for standard resolution or 120 × 0.2 mm for UHR, and a rotation time of 250 ms. All standard-resolution images were reconstructed using a slice thickness of 0.4 mm, a quantum iterative reconstruction (QIR) level of 3, a medium smooth kernel (Bv40), 70 and 55 keV, and a 512 × 512 reconstruction matrix [27]. When using UHR PCD-CT, a moderately sharp vascular convolution kernel was applied (Bv56, QIR level 3) after the best phase was determined [28].

### EID-CT acquisition protocol

Two single-source CT systems were used for the EID-CT cohort at the Semmelweis University Heart and Vascular Centre: a 256-slice (Philips iCT, Philips Healthcare) and a dedicated wide-detector cardiac CT scanner (GE CardioGraphe, GE Healthcare). Non-contrast scans utilised a tube voltage of 120 kVp and a tube current ranging from 15 to 50 mAs. All patients without contraindication received 0.8 mg of sublingual nitroglycerin. According to the patients’ anthropometrics, the following scan parameters were established for the CCTA: image acquisition was performed with rotation times of 270 ms and 240 ms. Systolic triggering was applied when the heart rate exceeded 75 beats per minute. Iomeprol contrast material (Iomeron 400, Bracco Ltd.) was administered through antecubital venous access at a flow rate of 4.5–5.5 mL/s with a four-phase protocol [29]. Proper scan timing was achieved using bolus tracking in the left atrium [8, 30]. Images were reconstructed with iDose level 4 or adaptive statistical iterative reconstruction level 70 [29].

Supplementary Table 1 and 2 list the key specifications of the imaging protocols for the three CT scanners used in the study.

### Coronary CT evaluation

Four readers with Level III certification and 5–10 years of experience in CCTA imaging evaluated the CCTA scans using a segment-based coronary tree model. Patients’ coronary calcium score was evaluated by the Agatston method on axial series using a dedicated semi-automated software tool (syngo.via, version VB60A) [31]. Coronary segments with a minimum of 1.5 mm diameter were evaluated for the segment-based analysis. Readers recorded lesion location and severity (semi-quantitative assessment, 1–24%: minimal, 25–49%: mild, 50–69%: moderate, 70–99%: severe, 100%: occlusion) and ≥ 50% or ≥ 70% stenosis category was defined for all patients, vessels, and segments. Vessel-based analysis was also carried out in each study population comprising the four main vessels: left main coronary artery, left anterior descending coronary artery (LAD), left circumflex coronary artery, and right coronary artery [32, 33].

### Invasive coronary angiography

All patients in the study were referred to ICA within 2 months based on their CCTA scan results as part of their clinical routine. ICA was conducted using a standardised technique by board-certified interventional cardiologists with more than 10 years of experience. A minimum of five projections of the left and right coronary systems were acquired in each patient. All coronary segments were analysed blinded to CCTA results, using a minimum of two projections. ICA reports comprised of the degree of stenosis (semi-quantitative assessment, 1–24%: minimal, 25–49%: mild, 50–69%: moderate, 70–99%: severe, 100%: occlusion) on a segmental basis and results were recorded in a structured reporting platform. Similarly, to the CT cohorts, the ≥ 50% or ≥ 70% stenosis category was defined. The results from these validated ICA reports were used for this study, and no additional study reading (quantitative coronary angiography) was performed. Anatomical mismatches were evaluated, and a consensus read was performed in 8 cases to confirm that the same lesion was quantified by CCTA and ICA.

### Simulation model for ICA referrals

To estimate the potential difference in ICA referrals, a previously designed simulation model was adjusted to only include CAD-RADS 3 and 4A patients. The model was described in detail previously [34]. In brief, the model utilised patient-level sensitivity, specificity, and predictive values for stenosis thresholds of ≥ 50% and ≥ 70%, obtained from the diagnostic accuracy analysis of the PCD-CT and EID-CT cohorts. A decision-tree framework was employed to simulate clinical decision-making pathways and subsequent ICA referrals based on these diagnostic metrics. Our Monte Carlo simulation adhered to guideline-based care pathways consistent with PROMISE. In the decision model, CAD-RADS 3 cases underwent functional testing, with ICA performed only if results were positive. By contrast, CAD-RADS 4A, 4B, or 5 cases were eligible for direct referral to ICA. Accordingly, ICA was modelled as either a direct consequence of the CAD-RADS category (4A and 4B/5) or an indirect consequence mediated by functional testing (3). Consequently, functional testing volumes are directly linked to ICA use—when ICA decreases, functional testing also decreases. Given that ICA is the most clinically relevant and auditable endpoint, it is reported as the primary outcome, while we frame the overall effect as a reduction in downstream evaluation (including functional testing-driven ICA) to align with guideline-based care. Monte Carlo simulations with 30,000 iterations (assumed number of patients over a 10-year scan period) were performed to account for inherent variability, and bootstrap analysis with 1000 resamples was conducted to estimate the robustness and variability of the ICA referral predictions between the two imaging modalities. The code of the Monte Carlo simulation model was evaluated by Python libraries, and it is available on reasonable request.

### Statistical analysis

Categorical variables were represented as counts with corresponding percentages in parentheses, while quantitative variables were expressed as means ± standard deviations or median and interquartile ranges [IQR]. To evaluate the diagnostic performance of the three different CT groups compared to the reference standard of ICA, we calculated sensitivity, specificity, positive predictive value (PPV), negative predictive value (NPV), and accuracy for detecting a stenosis of ≥ 50% and ≥ 70% on patient-, vessel- and segment-based levels (results reported with 95% confidence intervals (CIs)). Additionally, receiver operating characteristic curve analysis was conducted, and the area under the curve (AUC) was calculated [35]. We estimated the ESC 2024 guideline-based coronary artery calcium score-weighted clinical likelihood for all patients included in the study to ensure comparability between groups [36]. The calcium score-weighted clinical likelihood of obstructive CAD was computed with the published R function cadptp_cta provided by Winther et al, including sex, age, symptom category, number of risk factors, and CACS as inputs. Also, we report three clinical likelihood categories: low (< 15%), moderate (15–50%), and high (≥ 50%) based on the calculated calcium score-weighted clinical likelihood values per group. A subanalysis was performed for PCD-CT patients where the UHR scan mode was applied [37]. We used the Hanley & McNeil test to compare AUC values of independent patient populations based on the standard errors. Accuracy, sensitivity, and specificity values were compared using z-statistics based on the standard errors of each accuracy value. For the comparison of cardiovascular risk among the three CTA groups, a two-sample *t*-test was used for normally distributed parameters, while the Mann–Whitney *U*-test was applied for nonparametric data. A chi-square test was used for categorical data comparison. Statistical analysis was carried out using SPSS Statistics, version 29.0 (IBM) and MedCalc v 22.030.

## Results

### Patient, CCTA and ICA characteristics

In the PCD-CT population, a total of 143 patients (mean age 66 ± 9 years, 27.3% female) with 572 vessels and 2431 segments were evaluated. UHR-PCD-CT was performed in 55/143 (38.5%) patients. The median Agatston score was 773.0 [IQR: 272.5–1337.2]. A total of 63 (44.1%) patients had an Agatston score of ≥ 1000.

The PCD-CT patient characteristics are summarised in Table 1, and ICA characteristics in this group are summarised in Supplementary Table 3.

**Table 1:** Patient and CCTA characteristics

Characteristics	PCD-CT (*n* = 143)	EID-CT (*n* = 109)	*p*-value
Demographic characteristics			
Age (years)	66.4 ± 9.6	65.1 ± 9.6	0.51
Female sex, *n* (%)	39 (27.3)	31 (28.4)	0.84
BMI (kg/m^2^)	29.4 ± 6.3	28.3 ± 3.8	0.32
Cardiovascular risk factors			
Hypertension, *n* (%)	128 (89.5)	97 (88.9)	0.89
Hyperlipidemia, *n* (%)	106 (74.2)	83 (76.2)	0.71
Diabetes mellitus, *n* (%)	42 (29.4)	37 (33.9)	0.44
Smoking history, *n* (%)	43 (30.1)	46 (42.2)	0.05
CT findings			
Agatston score	773.0 [272.5-1337.2]	461.5 [206.0-1115.0]	0.01
Agatston score ≥ 1000, *n* (%)	63 (44.1)	61 (55.9)	0.06
Calcium score-weighted clinical likelihood of CAD	64.6%	65.3%	0.82
ICA findings			
1-vessel disease (≥ 50%)	74 (51.7)	57 (52.3)	0.49
2-vessel disease (≥ 50%)	29 (20.3)	22 (20.2)	
3-vessel disease (≥ 50%)	18 (12.6)	7 (6.4)	
LM and 3-vessel disease (≥ 50%)	8 (5.6)	4 (3.7)	

Categorical variables are presented as counts with percentages in parentheses, while quantitative variables are expressed as means ± standard deviations or as medians with IQRs in brackets

*BMI* body mass index, *CAD* coronary artery disease, *ICA* invasive coronary angiography, *IQR* interquartile range, *LM* left main coronary artery, *PCD-CT* photon-counting detector computed tomography, *EID-CT* energy-integrating detector computed tomography, *CCTA* coronary computed tomography angiography

The EID-CT group included 109 patients (65 ± 9 years, 31.0% female) with 436 vessels and 1853 segments (median Agatston score 461.5 [IQR: 206.0–1115.0]). The EID-CT patient characteristics are summarised in Table 1.

The PCD-CT and EID-CT groups had similar cardiovascular risk profiles, with the only difference being a higher prevalence of smoking in the EID-CT group, 46 (42.2%), compared to PCD-CT 43 (30.1%) (*p* = 0.05). The median Agatston score was significantly higher in the PCD-CT cohort 773.0 [IQR: 272.5–1337.2] vs the EID-CT group 461.5 [IQR: 206.0–1115.0] (*p* = 0.01). (Table 1). The calcium score-weighted clinical likelihood of obstructive CAD was 64.6% ± 24.8 in the PCD-CT group and 65.3% ± 20.5 in the EID-CT group (*p* = 0.82) (Table 1). Per the ESC 2024 guidelines, most patients in both cohorts categorised in the moderate or high pretest likelihood categories (PCD-CT: moderate 24/143 [16.8%], high 110/143 [76.9%]; EID-CT: moderate 24/109 [22.0%], high 84/109 [77.1%]), while few low-risk cases were detected (PCD-CT 9/143 [6.3%]; EID-CT 1/109 [0.9%]). The distribution of categories did not differ significantly between modalities (*p* = 0.067).

### Diagnostic performance of PCD-CT and UHR-PCD-CT

Vessel disease prevalence based on PCD-CT vs ICA was 33.4% vs 36.8% for ≥ 50% and 25.4% vs 23.1% for ≥ 70% stenosis, whereas segment-based disease prevalence was 8.3% vs 8.2% for ≥ 50% and 6.6% vs 5.6% for ≥ 70% stenosis, respectively.

The patient-based, vessel-based, and segment-based accuracy of PCD-CT in detecting a stenosis of ≥ 50% compared to ICA was 88.1% (95% CI: 81.6–92.9%); 91.6% (95% CI: 89.0–93.8%); 97.7% (95% CI: 97.0–98.3%), respectively. Regarding the detection of ≥ 70% stenosis by PCD-CT as compared with ICA, patient-based, vessel-based, and segment-based accuracy values were as follows: 90.9% (95% CI: 84.9–95.1%); 94.6% (95% CI: 92.4–96.3%); 98.6% (95% CI: 98.0–99.0%).

**Fig. 2:**
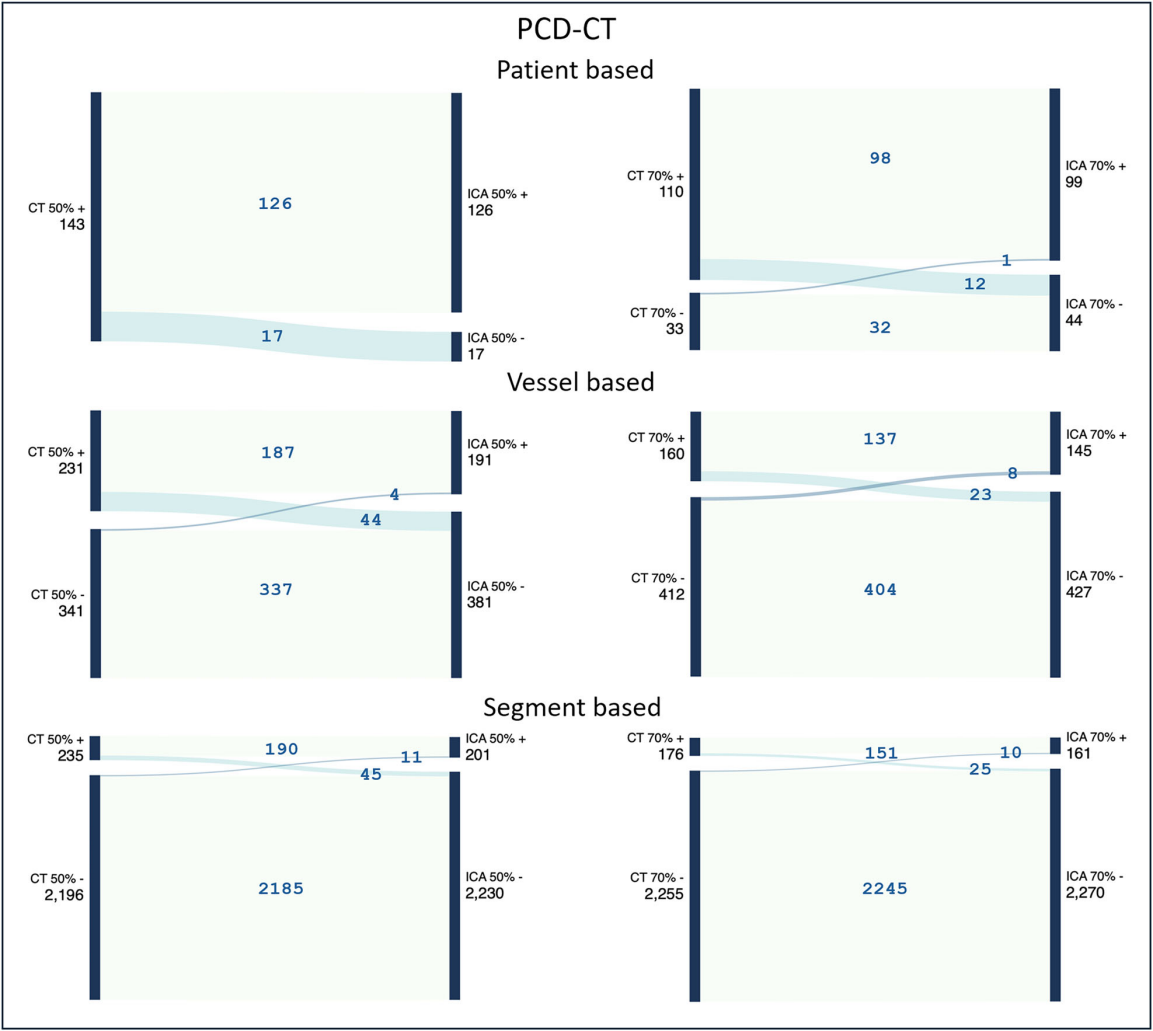
Flow of stenosis classification in PCD-CT based on ICA results. To better visualise the accuracy of PCD-CT in evaluating 50% and 70% stenosis, a Sankey diagram displays the stenosis classification based on patients, vessels, and segments, with ICA serving as the reference standard. PCD-CT, photon-counting detector computed tomography; ICA, invasive coronary angiography

**Fig. 3:**
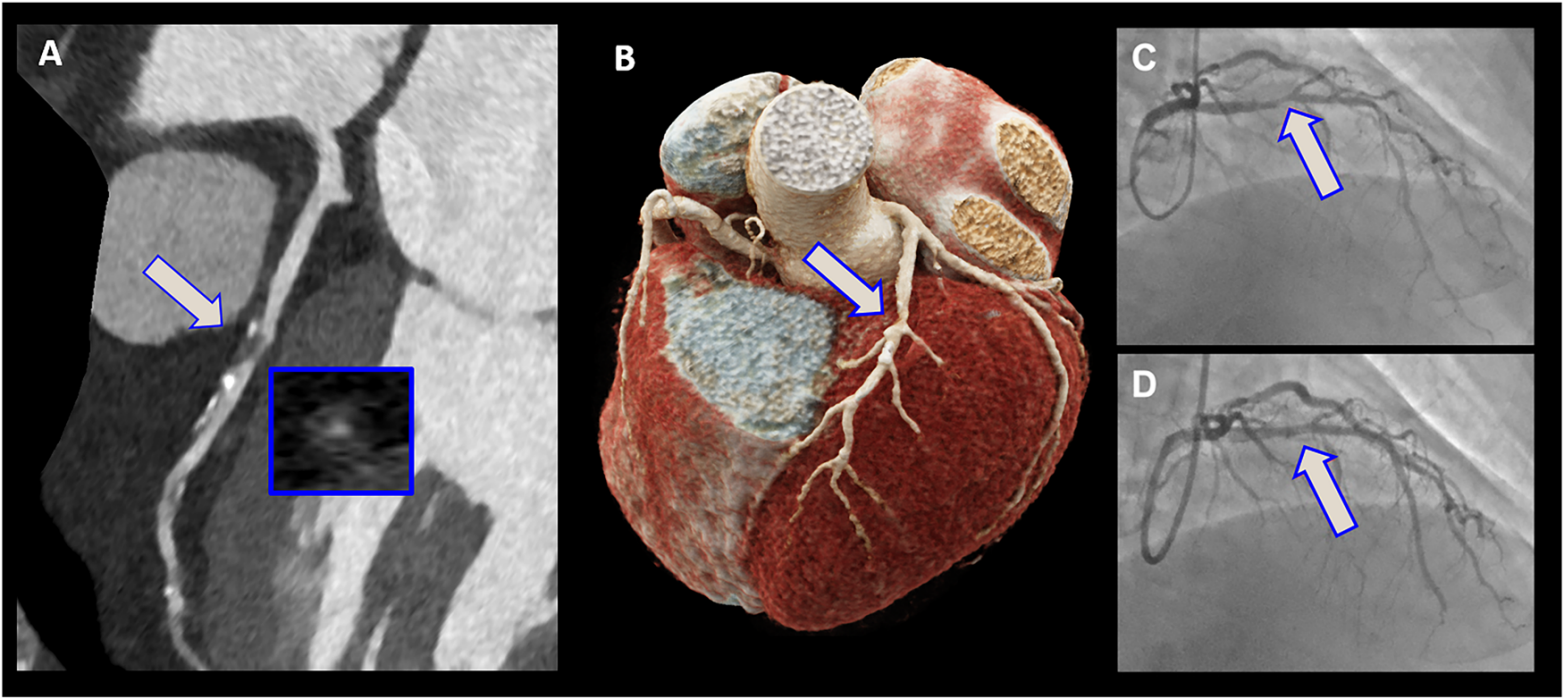
A representative case of PCD-CT for the detection of obstructive CAD. A 72-year-old male with stable chest pain was referred to PCD-CT examination. A ≥ 70% stenosis was detected on the proximal LAD. The patient was then referred to ICA, where a similar stenosis degree on the LAD was found, and PCI was carried out. Panel **A** shows the multiplanar reconstruction of the LAD, within the small, boxed square, the cross-sectional view can be seen at the stenosis location. **B** Displays the 3-dimensional view of the heart. **C** The ICA image of the LAD can be seen with ≥ 70% stenosis in the proximal segment, while **D** shows the LAD coronary artery after the successful stent implantation. ICA, invasive coronary angiography; PCD-CT, photon-counting detector computed tomography; LAD, left anterior descending coronary artery; CAD, coronary artery disease; PCI, percutaneous coronary intervention

Tables 2 and 3 summarise PCD-CT’s specificity, sensitivity, PPV, and NPV parameters detecting ≥ 50% and ≥ 70% stenosis. Figure 2 depicts the Sankey diagrams summarising the patient, vessel, and segment-based stenosis classification using ICA as the reference standard. Figure 3 displays a patient who underwent PCD-CT and subsequent ICA, and based on the ICA result, percutaneous coronary intervention was performed.

**Table 2:** Diagnostic performance of PCD-CT, UHR PCD-CT, and EID-CTs for the detection of ≥ 50% stenosis as compared to ICA

	PCD-CT total		UHR subgroup		EID-CT	
	≥ 50% stenosis					
	Value (%)	95% CI (%)	Value (%)	95% CI (%)	Value (%)	95% CI (%)
Patient based						
Sensitivity	100.0	97.1–100.0	100.0	92.9–100.0	100.0	95.8–100.0
Specificity	-	-	-	-	-	-
PPV	88.1	88.1–88.1	90.9	90.9–90.9	77.8	76.4–79.1
NPV	-	-	-	-	-	-
Accuracy	88.1	81.6–92.9	90.9	80.1–96.9	77.9	69.0–85.4
Vessel based						
Sensitivity	97.9	94.7–99.4	100.0	95.5–100.0	93.5	87.6–97.2
Specificity	88.5	84.8–91.5	87.1	80.3–92.1	71.6	66.2–76.5
PPV	80.9	76.3–84.9	81.8	74.5–87.4	56.4	51.9–60.8
NPV	98.8	96.9–99.6	100.0	97.0–100.0	96.6	93.5–98.2
Accuracy	91.6	89.0–93.8	91.8	87.4–95.1	77.8	73.6–81.6
Segment based						
Sensitivity	94.5	90.4–97.2	98.7	92.9–99.9	80.0	72.4–86.3
Specificity	97.9	97.3–98.5	98.6	97.6–99.3	93.5	92.2–94.6
PPV	80.9	75.9–84.9	86.4	78.3–91.8	50.0	45.1–54.9
NPV	99.5	99.1–99.7	99.8	99.1–99.9	98.3	97.6–98.8
Accuracy	97.7	97.0–98.3	98.6	97.6–99.3	92.4	91.2–93.6

Excellent vessel- and segment-based accuracy (> 90%) was found using PCD-CT for detecting ≥ 50% stenosis. EID-CT yielded lower patient, vessel and segment-based accuracy. Patient-based specificity and NPVs could not be calculated since no true or false negative cases were included in the cohort

Categorical variables are represented as counts with corresponding percentages in parentheses, while quantitative variables are expressed as means ± standard deviations

*CI* confidence interval, *EID* energy-integrating detector, *PCD* photon-counting detector, *UHR* ultra-high resolution, *ICA* invasive coronary angiography, *PPV* positive predictive value, *NPV* negative predictive value

**Table 3:** Diagnostic performance of PCD-CT, UHR PCD-CT, and EID CTs for the detection of ≥ 70% stenosis as compared to ICA

	PCD-CT total		UHR subgroup		EID-CT	
	≥ 70% stenosis					
	Value (%)	95% CI (%)	Value (%)	95% CI (%)	Value (%)	95% CI (%)
Patient based						
Sensitivity	98.9	94.5–99.9	96.9	83.8–99.9	90.5	81.5–96.1
Specificity	72.7	57.2–85.0	86.9	66.4–97.2	28.6	14.6–46.3
PPV	89.1	83.4–92.9	91.2	78.2–96.8	72.8	68.2–76.9
NPV	96.9	81.9–99.6	95.2	74.3–99.3	58.8	37.3–77.5
Accuracy	90.9	84.9–95.1	92.7	82.4–97.9	70.6	61.2–78.9
Vessel based						
Sensitivity	94.5	89.4–97.6	94.1	83.8–98.8	79.0	69.7–86.5
Specificity	94.6	92.0–96.6	96.5	92.4 –98.7	81.5	76.9–85.6
PPV	85.6	79.9–89.8	88.9	78.4–94.6	56.0	49.9–61.9
NPV	98.1	96.3–99.0	98.2	94.8–99.4	92.8	89.9–95.0
Accuracy	94.6	92.4–96.3	95.9	92.4–98.1	80.9	76.9–84.5
Segment based						
Sensitivity	93.8	88.9–96.9	96.2	86.8–99.5	65.7	55.9–74.6
Specificity	98.9	98.4–99.3	99.1	98.2–99.6	95.8	94.8–96.7
PPV	85.8	80.3–89.9	86.2	75.8–92.6	49.3	42.8–55.8
NPV	99.6	99.2–99.8	99.8	99.1–99.9	97.8	97.2–98.3
Accuracy	98.6	98.0–99.0	98.9	98.0–99.5	94.1	92.9–95.1

PCD-CT outperformed EID-CTs in detecting ≥ 70% stenosis, based on sensitivity, specificity and accuracy, at the vessel and segment levels. The UHR subgroup demonstrated similar diagnostic performance as the whole PCD-CT population, although the UHR mode was applied in patients with extensive coronary calcification

Categorical variables are represented as counts with corresponding percentages in parentheses, while quantitative variables are expressed as means ± standard deviations

*CI* confidence interval, *PCD-CT* photon-counting detector computed tomography, *EID-CT* energy-integrating detector computed tomography, *ICA* invasive coronary angiography, *PPV* positive predictive value, *NPV* negative predictive value, *UHR* ultra-high resolution

UHR mode was used in 55/143 patients (38.5%). In a subanalysis, we evaluated the accuracy, specificity, sensitivity, PPV and NPV parameters of UHR PCD-CT in detecting a stenosis of ≥ 50% and ≥ 70% on ICA (Table 3*)*. Supplementary Fig. 1 shows a representative case of a patient who underwent UHR PCD-CT and ICA.

### Diagnostic performance of conventional EID-CT

In the EID-CT cohort, the vessel-based disease prevalence was 28.2% for ≥ 50% and 22.9% for ≥ 70% stenosis, while the segment-based disease prevalence was 7.6% for ≥ 50% and 5.8% for ≥ 70% stenosis on ICA, respectively. Tables 2 and 3 summarise the accuracy, specificity, sensitivity, PPV, and NPV of the EID-CT cohort.

### Comparison of PCD-CT vs EID-CT

The accuracy of PCD-CT in detecting ≥ 50% stenosis compared to ICA was significantly higher than that of EID-CT across all levels of analysis. On a patient level, PCD-CT achieved an accuracy of 88.1% (95% CI: 81.7–92.9%), whereas EID-CT reached 77.9% (95% CI: 69.0–85.4%). On a vessel level, PCD-CT demonstrated an accuracy of 91.6% (95% CI: 89.0–93.8%), outperforming EID-CT, which had an accuracy of 77.8% (95% CI: 73.6–81.6%). Finally, on a segment level, PCD-CT achieved the highest accuracy at 97.7% (95% CI: 97.0–98.3%), compared to 92.4% (95% CI: 91.2–93.6%) for EID-CT. Similarly, we detected significantly higher vessel- and segment-based sensitivity, specificity, and accuracy using PCD-CT as compared with EID-CT for ≥ 70% stenosis (*p* < 0.01, all comparisons).

Supplementary Fig. 2 depicts the Sankey diagrams summarising the patient, vessel, and segment-based stenosis classification using ICA as the reference standard.

Supplementary Fig. 3 displays the vessel and segment-based AUC analysis comparing the performance of EID-CT vs PCD-CT.

### Results of the Monte Carlo simulation model

During 30,000 simulation iterations, the total number of patients categorised as CAD-RADS 3 was similar for both modalities (PCD-CT: 2313.3 ± 51.0, EID-CT: 2308.3 ± 62.7), as was the total number of patients categorised as CAD-RADS 4 A (PCD-CT: 1435.3 ± 46.2, EID-CT: 1423.5 ± 43.4), indicating comparable baseline classification between groups. The Monte Carlo simulation, based on diagnostic accuracy parameters of both CT modalities, demonstrated a potential 14.8% mean reduction in ICA referrals when using PCD-CT compared to EID-CT, corresponding to a simulated ICA utilisation of 821.7 ± 28.0 for PCD-CT vs 964.3 ± 25.1 for EID-CT (*p* < 0.001).

## Discussion

Our study evaluated the diagnostic performance of PCD-CT and UHR-PCD-CT in symptomatic patients vs EID-CT for detecting ≥ 50% and ≥ 70% stenosis, using ICA as the reference. The results showed that PCD-CT demonstrated excellent vessel- and segment-based performance for stable angina patients referred for CCTA and yielded significantly higher patient-level accuracy than EID-CT. In patients with diffuse coronary calcification, the UHR mode also exhibited excellent diagnostic performance. Furthermore, as a novelty so far in the scientific literature regarding PCD-CT, in our study, we employed a decision and simulation model, which demonstrated a notable reduction in ICA utilisation— directly or indirectly by following also functional testing in patients with CAD-RADS 3 or 4—in favour of PCD-CT.

Advancements in CT technology have led to the development of the first commercially available dual-source PCD-CT, which is increasingly being incorporated into clinical practice. It utilises novel energy-resolving X-ray detectors that operate differently from traditional EIDs, leading to improved IQ, surpassing the capabilities of traditional CT technology [38]. Blooming artefacts in patients with extensive calcification are the main limitation of CT imaging, resulting in reduced specificity [39]. PCD-CT, with its improved resolution, can mitigate these issues, promising a more accurate assessment of the clinical likelihood of CAD. PCD-CT was associated with more accurate measurements of the reference than EID-CT, and importantly, it measured significantly lower values in the phantom and in patients, leading to a reclassification of 5.25% of the simulated patient cohort into a lower classification class [40]. The first-in-human results on IQ of PCD-CT were published by Si-Mohamed et al, in which it was demonstrated that PCD-CT showed enhanced IQ and increased diagnostic confidence in humans compared to EID-CT [41]. Since then, several studies have been reported in the scientific literature about PCD-CT’s positive aspects, although these studies are mainly phantom-based research results [39, 42]. Notably, Koons et al found that PCCT provides a more accurate assessment of coronary luminal stenosis than EID-CT, especially for ring-shaped plaques in a phantom setting [17]. A recently published phantom study showed reduced blooming artefacts, thus enhanced stenosis quantification accuracy, independent of heart rate, with UHR PCD-CT compared to standard resolution mode [20]. However, data on the diagnostic performance of novel PCD-CT systems—particularly in comparison with EID-CT—in real-life patient cohorts referred for CCTA are limited. In a previous retrospective study, 401 EID-CT and 411 PCD-CT scans were evaluated to assess the ICA recommendation rates based on CT findings [43]. In patients who underwent PCD-CT, despite the higher total Agatston score, the number of ICA recommendations was significantly lower than the number of EID-CT-based ICA referrals. The use of EID-CT was linked with increased recommendation of ICA in the overall patient population, in patients with an Agatston score of < 400 and in patients with ≥ 400 Agatston score. While these results demonstrate that PCD-CT can improve clinical decision-making, it is important to note that only the number of ICA recommendations was included in the analysis and not the results of ICAs.

Recently, a retrospective study by Sakai et al, including 926 vessels from 372 patients in the EID-CT group and 760 vessels from 285 patients in the PCD-CT group, evaluated the diagnostic performance of PCD-CT vs EID-CT using ICA as a reference standard. On a vessel-level analysis based on 1686 vessels, PCD-CT outperformed EID-CT in specificity, PPV, and overall diagnostic accuracy for detecting more than 50% stenosis. However, the sensitivity and NPV of obstructive CAD were similarly high between modalities, indicating that both modalities reliably exclude significant CAD, but PCD-CT offered superior specificity and precision in positive cases [44]. The 98.9% of patients were scanned with standard scan mode, and UHR was not utilised. Notably, more than 50% stenosis based on quantitative coronary angiography was found only in 107 vessels (11.6%) in the PCD-CT group and in 77 vessels (10.1%), and the median Agatston scores were 5.0 (0.0–134.0) for EID-CT and 8.0 (0.0–144.0) for PCD-CT, reflecting a low-risk patient population. We analysed a comparable number of segments (1008 vessels) but also applied UHR mode (in 38.5% of cases) for heavily calcified vessels with a median Agatston score of 773 [272.5–1337.2] and evaluated the accuracy of detecting ≥ 70% stenosis. Additionally, our analysis provides a more detailed aspect, as not only vessel-based, but also segment-based evaluations were calculated, demonstrating excellent performance of PCD-CT, which strengthens its clinical value. Also, we demonstrate the benefits of PCD-CT in patients with substantially larger Agatston scores than EID-CT or other patient cohorts, and despite higher vessel-based disease prevalence of ≥ 50% (33.4%) or ≥ 70% (25.4%) stenosis. Furthermore, as a novelty so far in the scientific literature regarding PCD-CT, in our study, we employed a decision and simulation model, which demonstrated a notable reduction in ICA utilisation—following also functional testing in patients with CAD-RADS 3 or 4—in favour of PCD-CT. In another clinical study comprised of 68 high-risk patients with severe aortic valve stenosis, UHR-PCD-CT was evaluated to assess diagnostic performance. CAD was defined as stenosis of 50% or greater, and the results of the validated ICA reports served as a reference. Using UHR mode, the accuracy was 88%, and the AUC was 0.93 for detecting obstructive CAD. Notably, this patient population was older (81 ± 7 years), had lower BMI (26.6 ± 4.5), lower rates of smoking or diabetes mellitus as compared with the current study populations. The median Agatston score was 414 [IQR: 125–1246], and 44 out of 68 patients (65% of all included patients) had no ≥ 50% stenosis present. The disease prevalence of CAD for both vessel- and segment-based analysis was lower despite the PCD-CT being performed as part of TAVR planning in high-risk patients. Our patient cohort is characterised by extensive coronary calcification, high rates of traditional cardiovascular risk factors, elevated BMI, and higher stenosis grade (76.6% of patients had any coronary stenosis ≥ 70%). Despite the more advanced coronary atherosclerosis, we found even higher accuracy for detecting ≥ 70% stenosis between CCTA and ICA. Our subanalysis, using UHR imaging, further reinforced previous findings and demonstrated exceptional diagnostic performance for detecting ≥ 70% luminal narrowing. Moreover, our analysis also included two more patient cohorts derived from two different EID-CT scanners with similar cardiovascular risk profiles and disease prevalence. Compared to EID-CT, PCD-CT demonstrated superior performance based on the significantly higher sensitivity, specificity, accuracy, and AUC values, possibly resulting from the aforementioned benefits of higher resolution and improved IQ. Our results strengthen the previous findings suggesting improved stenosis assessment using PCD-CT as compared with EID-CT, ultimately enhancing patient management through improved diagnostic capabilities and the potential to safely exclude obstructive CAD in higher-risk individuals. The Monte Carlo simulation performed in this study revealed a potential mean reduction of 14.8% in ICA referrals using PCD-CT compared to EID-CT. By lowering the reliance on ICA, the associated risks and potential complications are also diminished, contributing to improved patient safety. Additionally, this shift in practice alleviates the workload on healthcare providers and reduces resource strain, resulting in more efficient management of patient care and easing the overall burden on healthcare systems.

We acknowledge the limitations of our study. First, it had a small sample size in a three-centre analysis, although there are currently no data on this patient cohort, and the size of the population is similar to other published studies on PCD-CT assessing IQ or spectral capabilities. Furthermore, we acknowledge that our study is not a directly self-controlled study, although due to radiation exposure risks, we refrained from performing both PCD-CT and EID-CT in the same patients. Moreover, retrospective comparisons allow for larger, more representative cohorts, that reflect true clinical decision-making pathways, enabling better-powered diagnostic accuracy analyses. Our group previously conducted an intra-individual comparison [24] that demonstrated the higher accuracy of PCD-CT compared to QCA and showed that PCD-CT assigns nearly half of patients to a lower CAD-RADS category. While this design offers strong internal validity, it is not feasible for larger cohorts due to ethical concerns and patient reluctance to undergo two CT scans. This is why we opted for a larger, multicentre study using comparable cohorts, to better reflect real-world clinical practice and evaluate the potential of PCD-CT to reduce unnecessary ICA referrals. Second, in our study, the cut-off Agatston-score for using UHR mode was 300 early after the introduction of UHR, although there is no guideline regarding the proper use of this mode, and it is not used consistently by other centres. Moreover, spectral imaging with higher VMI-s [23] demonstrated further improvement and smaller bias compared to ICA, these were not used in our study for the standard resolution images. Therefore, further studies are needed to determine whether the UHR mode is more accurate above a certain Agatston score. Third, we did not refer patients with non-obstructive CAD ( < 50% stenosis) for invasive angiography, as there was no equipoise given that this practice is against current guidelines. Fifth, a quantitative analysis of ICA was not performed.

The Dual Source PCD-CT scanner provides excellent diagnostic performance for the identification of obstructive CAD. Compared with EID-CT, PCD-CT demonstrated superior diagnostic accuracy, which translated into a simulated reduction in the need for further downstream evaluation. Our findings suggest that the improved accuracy of PCD-CT may reduce unnecessary referrals to ICA, thereby enabling more efficient, patient-centred care and alleviating healthcare system burden.

## Supplementary information

ELECTRONIC SUPPLEMENTARY MATERIAL

## Abbreviations

CAD: Coronary artery disease
CCTA: Coronary CT angiography
EID: Energy-integrating detector
ICA: Invasive coronary angiography
IQ: Image quality
IQR: Interquartile range
LAD: Left anterior descending coronary artery
NPV: Negative predictive value
PCD: Photon-counting detector
PPV: Positive predictive value
UHR: Ultra-high resolution
