# Supplementary material for: Comparative analysis of photon-counting and energy-integrating detector CT to identify obstructive coronary artery disease
Source: Eur Radiol. 2025 Nov 14;36(4):3091–102. doi: 10.1007/s00330-025-12118-7 (PMC13035877; doi:10.1007/s00330-025-12118-7)
Supplement: Supplementary file 1 — ELECTRONIC SUPPLEMENTARY MATERIAL [file 330_2025_12118_MOESM1_ESM.pdf]

Comparative Analysis of Photon-Counting and Energy-Integrating Detector CT to Identify Obstructive Coronary Artery Disease

ELECTRONIC SUPPLEMENTARY MATERIAL

Supplementary Figure 1. A representative case showing a 70% stenosis on the mid-right coronary artery.

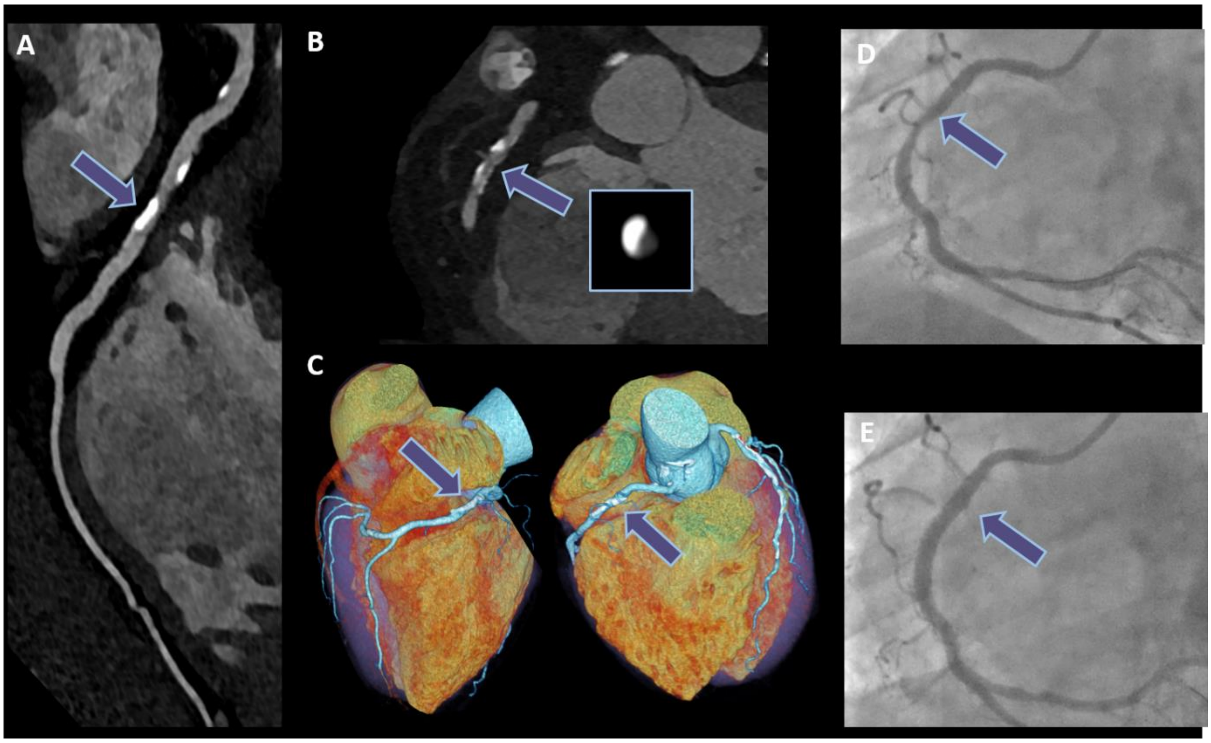

A 65-year-old male with stable chest pain was referred to PCD-CT examination. Due to extensive calcification the UHR mode was applied and a ≥70% stenosis was detected on the RCA. The patient was then referred to ICA, where a similar stenosis degree on the RCA was found, and PCI was carried out. Panel A shows the multiplanar reconstruction of the RCA, while panel B represents the c-view of the RCA. Within the small, boxed square the cross-sectional view can be seen at the stenosis location. Panel C shows the 3-dimensional view of the heart. In panel D, the ICA image of the RCA can be seen with ≥70% stenosis in the middle segment, while panel E shows the RCA after the successful stent implantation.

Abbreviation: ICA: invasive coronary angiography, PCD-CT: photon-counting detector computed tomography, RCA: right coronary artery, UHR: ultra-high-resolution

Supplementary Figure 2. Sankey diagram of EID-CT stenosis classification based on ICA.

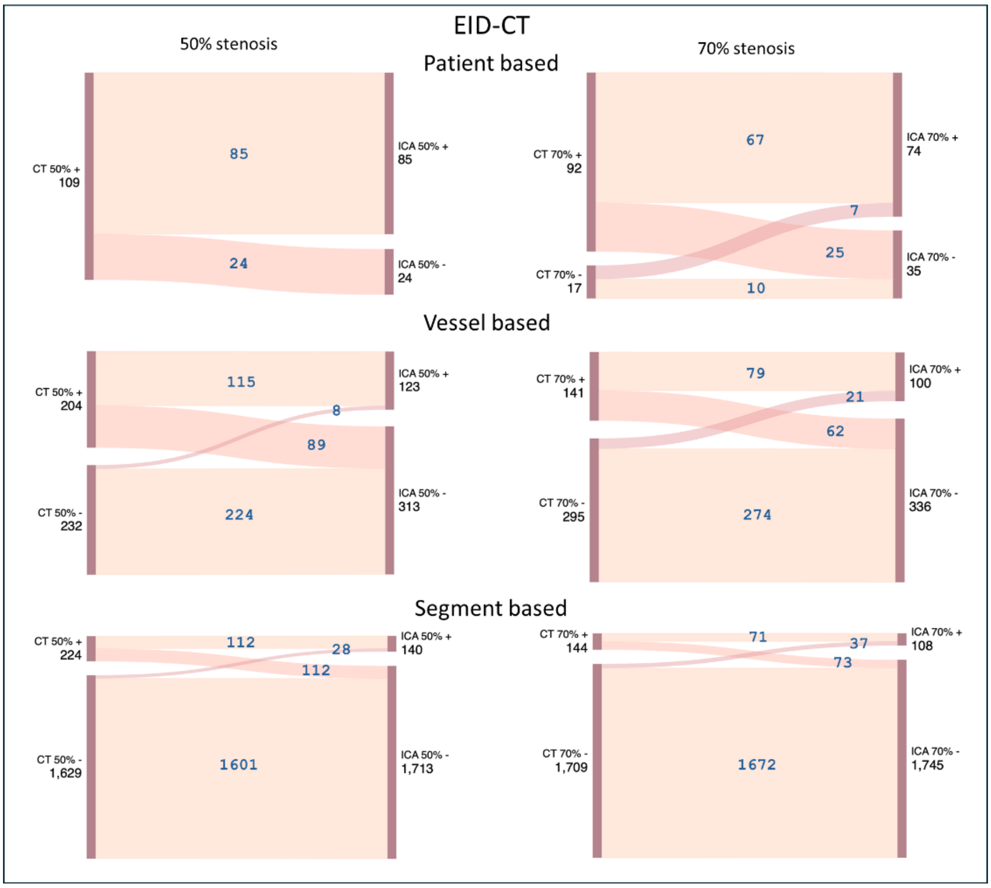

Sankey diagrams illustrate the performance of EID-CT in assessing ≥50% and ≥70% stenosis. The figure depicts the classification of stenosis categories at the patient, vessel, and segment levels, using ICA as the reference standard.

Abbreviation: EID: energy-integrating detector, ICA: invasive coronary angiography

Supplementary Figure 3. ROC curves for vessel and segment-based analysis comparing the performance of EID-CT vs PCD-CT.

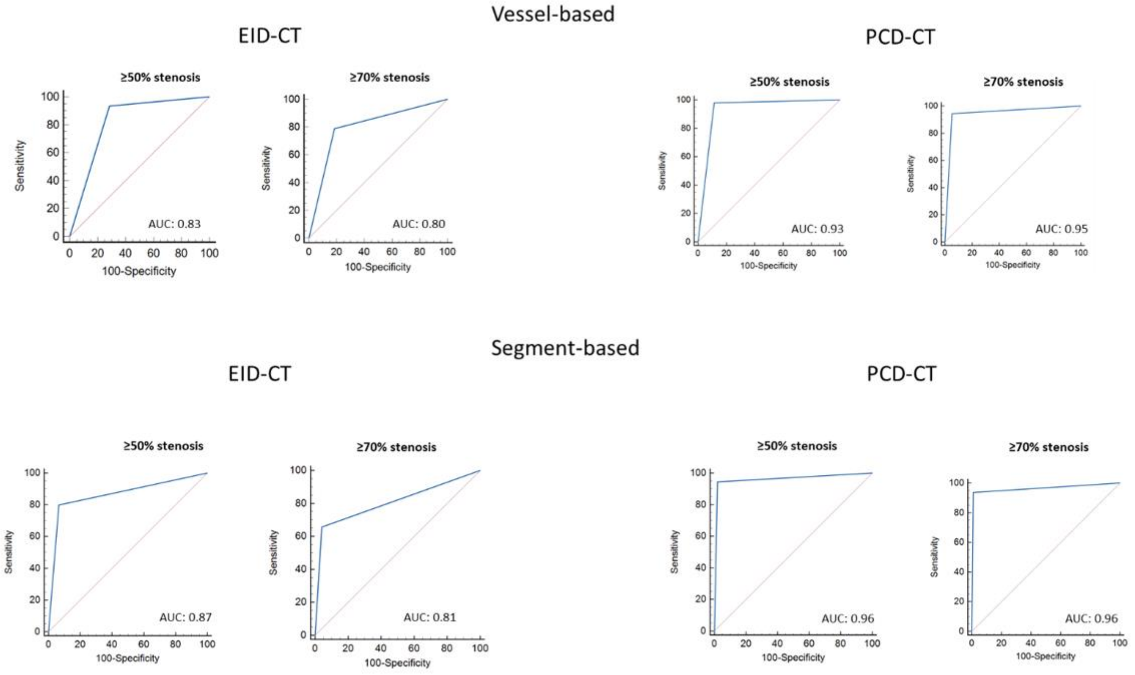

Receiver operating characteristic curves demonstrate a higher AUC for PCD-CT compared to EID-CT in detecting ≥50% and ≥70% stenosis**.**

Abbreviation: AUC: Area Under the Curve, EID: energy-integrating detector, ROC: receiver operating characteristics, PCD-CT: photon counting detector CT

Supplementary Table 1. Imaging and reconstruction protocol

	PCD-CT Siemens NAEOTOM Alpha		EID-CT	
	Standard	UHR	Philips iCT	GE Cardiographe
**Tube voltage (kVp)**	120/140	120	100/120	100/120
**Collimation (mm)**	144 × 0.4	120 × 0.2	128 × 0.625	280 × 0.5
**Gantry rotation time (ms)**	250	250	270	240
**Temporal resolution (ms)**	66	66	135	120
**Z-axis coverage (cm)**	5.7	2.4	8	14
**Matrix size**	512 × 512	512 × 512	512 × 512	512 × 512
**Slice thickness (mm)**	0.4	0.2	0.625	0.5
**Iterative reconstruction**	QIR 3	QIR 3	iDose 4	ASIR 70

Abbreviation: ASIR: advanced adaptive statistical iterative reconstruction, EID-CT: Energy-integrating detector CT, PCD-CT: photon-counting detector computed tomography, QIR: quantum iterative reconstruction, UHR: ultra-high resolution

Supplementary Table 2. PCD-CT and invasive coronary angiography characteristics

Characteristics	PCD-CT (n=143)	EID-CT (n=109)	p-value
Contrast material (ml)	83.9 ± 7.7	90.1 ± 5.9	< 0.001
Flow rate (ml/min)	5.0 ± 0.4	4.9 ± 0.5	< 0.001
Heart rate (beats/min)	64.1 ± 9.6	63 ± 13.5	0.422
Total DLP (mGy*cm)	432.9 ± 311.2	383.7 ± 152.2	0.136
Sequential scan mode	89 (62.2%)	79 (72.5%)	0.116
140 kVp usage	89 (62.2%)	-	-
UHR scan mode	55 (38.5%)	-	-

Categorical variables are represented as counts with corresponding percentages in parentheses, while quantitative variables are expressed as means ± standard deviations.

Abbreviations: DLP: dose length product, PCD-CT: Photon-counting detector CT, UHR: ultra-high resolution

Supplementary Table 3. Invasive Coronary Angiography Characteristics in the PCD-CT group

**ICA Characteristics**	n=143
Contrast material (ml)	93.8 (66.2)
Heart rate (beats/min)	73.5 (14.7)
Radiation time (min)	6.6 (7.3)
Radiation dose (mGy)	689.3 (984.9)
Total DAP (Gy*cm^2)	52.9 (71.6)
Number of PCI, n (%)	97 (67.8)
Number of PCI in case of ≥ 50% stenosis on CCTA, n	97 out of 143 patients
Number of PCI in case of ≥ 70% stenosis on CCTA, n	96 out of 143 patients

Abbreviations: CCTA: coronary CT angiography, DAP: dose area product, ICA: invasive coronary angiography, PCI: percutaneous coronary intervention
